# History and Future of HER2-Targeted Therapy for Advanced Gastric Cancer

**DOI:** 10.3390/jcm12103391

**Published:** 2023-05-10

**Authors:** Shin Ariga

**Affiliations:** Department of Medical Oncology, Faculty of Medicine and Graduate School of Medicine, Hokkaido University, Sapporo 060-8638, Japan; s-ariga@med.hokudai.ac.jp

**Keywords:** gastric cancer, HER2, targeted therapy, heterogeneity

## Abstract

Human epidermal growth factor receptor 2 (HER2) is a receptor tyrosine kinase that belongs to the human epidermal growth factor receptor family. It is overexpressed/amplified in approximately 20% of gastric or gastroesophageal junction cancers. HER2 is being developed as a therapeutic target in a variety of cancers, and several agents have been shown to be effective in breast cancer. The development of HER2-targeted therapy for gastric cancer successfully began with trastuzumab. However, while effective in breast cancer, the successive anti-HER2 agents lapatinib, T-DM1, and pertuzumab failed to demonstrate benefits regarding survival in gastric cancer compared with the existing standard therapies. Intrinsic differences lie between gastric and breast cancer in terms of HER2-positive tumor biology, which may make development difficult. Recently, a novel anti-HER2 agent, trastuzumab deruxtecan, was introduced, and the development of HER2-positive gastric cancer agents has been moving to the next stage. This review summarizes the current HER2-targeted therapy for gastric or gastroesophageal cancer in chronological order and describes the promising future of HER2-targeted therapy.

## 1. Introduction

Advances in novel drugs and sequencing technologies have made biomarker-based, personalized, and effective treatment options available for patients with various types of solid tumor. Biomarker-based targeted therapy has dramatically changed the treatment of various cancers, such as non-small cell lung cancer [1]. The first biomarker to be developed as a therapeutic target in gastric adenocarcinoma and gastroesophageal junction adenocarcinoma (hereafter, gastric cancer) was human epidermal growth factor 2 (HER2).

HER2 belongs to the human epidermal growth factor receptor (EGFR) family of tyrosine kinase receptors. HER2 has no activating ligand but can undergo dimerization with EGFR family members to activate multiple downstream signaling pathways, including the RAS/RAF/MAPK and PI3K/AKT cascades [2]. The extracellular domain of HER2 consists of four subdomains (I–IV), including domain II, the so-called dimerization domain, which is essential for ligand-induced heterodimerization.

The frequency of HER2 overexpression/amplification in gastric cancer is approximately 20% [3]. HER2 positivity is more common in gastroesophageal cancer as the primary site, and more common in intestinal-type tumors pathologically. However, the evaluation of HER2 as a prognostic factor remains controversial [4]. Although the efficacy of HER2-targeted therapies has been successfully demonstrated in breast cancer with HER2 overexpression/amplification, the development of HER2-targeted therapy for gastric cancer has not gone smoothly. This review first summarizes the history of major clinical trials of HER2-targeted therapy for advanced gastric cancer, then lists the difficulties encountered in HER2-targeted therapy for gastric cancer, focusing on the promising novel anti-HER2 agent trastuzumab deruxtecan, and finally summarizes ongoing the clinical trials of novel anti-HER2 therapy.

## 2. History of HER2-Targeted Therapy for Gastric Cancer

The development of HER2-targeted therapy for gastric cancer, akin to breast cancer, has been initiated with trastuzumab. Following the successful publication of the phase 3 trial of trastuzumab in 2009, landmark clinical trials appraising diverse HER2-targeted agents have been published (Table 1).

### 2.1. Trastuzumab

Trastuzumab was the first anti-HER2 monoclonal antibody and was developed in 1990 [5,6]. It binds to extracellular domain IV of HER2 and exerts antitumor activities via several mechanisms, including the inhibition of HER2-mediated signaling, the prevention of the cleavage of the extracellular domain of HER2, and the induction of antibody-dependent cellular cytotoxicity, but the exact mechanisms remain unknown [7]. The administration of trastuzumab concomitantly with chemotherapy was discovered to enhance the survival rate of patients afflicted with metastatic breast cancer that is positive for HER2 in 2001, and following that, trastuzumab improved outcomes in HER2-positive early-stage breast cancer in the adjuvant setting in 2005 [8,9]. In xenograft models, trastuzumab demonstrated efficacy regarding the inhibition of the growth of HER2-amplified gastric cancer and exhibited synergistic effects in combination with cytotoxic agents [10]. Against this background, the ToGA (Trastuzumab for Gastric Cancer) study, the first prospective study to examine the effect of HER2-targeting therapy in gastric cancer, was conducted [11].

This study was an open-label, multicenter, international, phase 3, randomized controlled trial undertaken in Asia, Central and South America, and Europe. HER2-positive advanced gastric cancer patients were randomly chosen, at a 1:1 ratio, to receive a chemotherapy regimen consisting of cisplatin 80 mg/m^2^ every 3 weeks plus fluoropyrimidine (fluorouracil 800 mg/m^2^ every 24 h intravenously for 5 days or capecitabine 1000 mg/m^2^ twice a day for 14 days) for six cycles, or the same chemotherapy in combination with trastuzumab for six cycles followed by trastuzumab monotherapy. Crossover to trastuzumab at the time of disease progression was not allowed. The primary endpoint was overall survival. Because there were no exploratory clinical trials before the ToGA study, the definition of “HER2-positive” gastric cancer was determined in this study. The HER2 status was evaluated using immunohistochemistry (IHC) and fluorescence in situ hybridization (FISH) for breast cancer. However, unlike breast cancer, HER2 staining is heterogeneous in gastric cancer, and hence the 10% cutoff value for breast cancer was not applied to the biopsy specimens. In addition, because the HER2 receptor in gastric cancer is mainly expressed on the lateral and basal membrane side of the cell, staining only on the basolateral or lateral membrane was also defined as HER2 cell membrane staining. The FISH cut-off value (HER2/CEP17 ratio) was set at ≥2.0, the same as the 2005 criteria for breast cancer. Patients were eligible for this study if their IHC screening scored 3+ or if FISH was positive. A total of 3665 patients were successfully screened using IHC or FISH, and 810 patients were determined to be “HER2-positive”. Finally, 584 patients were included in the primary analysis (*n* = 294; *n* = 290). As a result, trastuzumab plus chemotherapy significantly improved overall survival compared with chemotherapy alone (13.8 months vs. 11.1 months; hazard ratio 0.74; *p* = 0.0046). Importantly, post hoc analysis indicated that trastuzumab plus chemotherapy seemed to have no overall survival benefit in patients with a low expression of HER2 protein (IHC 0 or 1+), even though FISH was positive (10.0 months vs. 8.7 months; hazard ratio 1.07). Based on these results, “HER2-positive” gastric cancer has been considered as IHC 3+ or IHC 2+/FISH+. However, it should be noted that this definition is based on an exploratory analysis and that the effect of trastuzumab on IHC 3+/FISH gastric cancer cannot be determined using this study.

Not only was this study the first to demonstrate the efficacy of a molecular targeted drug in chemotherapy for gastric cancer, it was of great significance that precision medicine had been introduced in chemotherapy for gastric cancer. In breast cancer, following the development of trastuzumab, new anti-HER2 target agents had been producing results one after another, and it was expected that gastric cancer would become like breast cancer in terms of available therapies.

### 2.2. Lapatinib

Lapatinib is an oral tyrosine kinase inhibitor that targets HER1 and HER2. In HER2-positive breast cancer, the co-administration of lapatinib with capecitabine has been found to extend median progression-free survival compared to the administration of capecitabine alone following trastuzumab-based therapy (8.4 months vs. 4.4 months; hazard ratio 0.49; *p* < 0.001). In 2013, the results of two phase 3 clinical trials of lapatinib in combination with chemotherapy for HER2-positive gastric cancer, TyTAN and TRIO-013/LOGiC, were published.

TyTAN was the first randomized study to compare the efficacy and safety of lapatinib in the second-line treatment of HER2-positive advanced gastric cancer [12]. This was an open label phase 3 study conducted in Asia (mainland China, Japan, South Korea, and Taiwan). Patients were randomly assigned, at a 1:1 ratio, to a group administered with lapatinib at 1500 mg once per day plus paclitaxel at 80 mg/m^2^ on days 1, 8, and 15 of a 4-week cycle, or paclitaxel at 80 mg/m^2^ only on the same schedule. In this study, HER2 FISH positivity (HER2/CEP17 ratio ≥ 2.0) was considered as “HER2-positive”, regardless of IHC findings. The primary endpoint was overall survival. In total, 261 patients were randomly assigned to this study, of whom 65% were HER2 IHC 2/3+. Only 6% of patients had previously received trastuzumab. The median overall survival was 11.0 months with lapatinib plus paclitaxel, versus 8.9 months with paclitaxel alone, which was not significantly different (hazard ratio 0.84; *p* = 0.1044).

TRIO-013/LOGiC was a multicenter, double-blind, randomized study conducted in 22 countries in Asia, Europe, North America, and South America in order to evaluate the efficacy of lapatinib in the first-line setting [13]. Patients were randomly assigned at a 1:1 ratio to a group administered with CAPOX (oxaliplatin at 130 mg/m^2^ on day 1 for up to eight cycles and capecitabine at 1700 mg/m^2^ from days 1 to 14) plus lapatinib at 1250 mg, or with CAPOX plus placebo, in 21-day cycles.

IHC 3+ or HER2 amplification via chromogenic or silver in situ hybridization (ISH) was permitted in this study, but based on breast cancer data, only HER2 FISH positivity (HER2/CEP17 ratio ≥ 2.0) was considered as “HER2-positive” and included in efficacy analyses. The primary endpoint was the overall survival of patients with centrally confirmed HER2 FISH-positive disease. A total of 545 patients were randomly assigned as the intent-to-treat population and 487 patients were confirmed as having a HER2-positive status, of whom 83% were HER2 IHC 2/3+. The primary endpoint was 12.2 months in the lapatinib arm and 10.5 months in the placebo arm, which was not significantly different (hazard ratio 0.91; *p* = 0.3292). Neither study met the primary endpoint of overall survival.

### 2.3. T-DM1

Antibody–drug conjugates (ADCs) are highly targeted biopharmaceutical drugs that combine monoclonal antibodies with a cytotoxic payload via a chemical linker. Because the ADC binds with strong affinity to the antigen present on cells, it is possible to selectively deliver cytotoxic agents to the target tumor cells. Although the concept of ADC is old, and was first presented by Paul Ehrlich approximately 100 years ago, ADCs are currently among the fastest growing anticancer drugs [14].

Ado-trastuzumab emtansine (T-DM1) is an ADC consisting of trastuzumab covalently linked to the highly potent microtubule inhibitory drug emtansine via a stable thioether linker. The EMILIA study showed that T-DM1 significantly prolonged progression-free and overall survival compared with lapatinib plus capecitabine in HER2-positive advanced breast cancer previously treated with trastuzumab [15]. In 2013, based on the EMILIA study, T-DM1 received FDA approval for the treatment of patients with HER2-positive metastatic breast cancer, and was the first antibody–drug conjugate to receive full approval for solid cancer.

Followed by breast cancer, a phase 2/3 clinical trial of T-DM1 for HER2-positive gastric cancer, the GATSBY study, was conducted and the results were published in 2016 [16]. This study was an open-label, adaptive phase 2/3 trial assessing the effectiveness of T-DM1 against a taxane (docetaxel or paclitaxel) in individuals with HER2-positive advanced gastric cancer who had received prior treatment. To save time and cost, the GATSBY study had a seamless design, from the dose-finding phase 2 to the confirmatory phase 3. In the first stage, participants were assigned at random (2:2:1) to receive T-DM1 at a dosage of 3.6 mg/kg every three weeks, T-DM1 at a weekly dosage of 2.4 mg/kg, or a taxane regimen. Phase 2 examined two dosing regimens of T-DM1 for phase 3. Based on the data of the interim analysis, the independent data monitoring committee selected T-DM1 at 2.4 mg/kg weekly as the dose to proceed to stage 2. In stage 2, patients were randomly assigned (2:1) to groups receiving either T-DM1 (2.4 mg/kg weekly) or a taxane. Phase 3 of the study included patients from both stage 1 and stage 2. In this study, “HER2-positive” was defined as HER2 IHC 2+ and ISH-positive or IHC 3+, regardless of ISH, which is currently used in clinical practice based on the ToGA study data. The primary endpoint was overall survival. Patients were enrolled from 28 countries worldwide. The median overall survival was 7.9 months in the T-DM1 group and 8.6 months in the taxane group (HR 1.15; one-sided *p* = 0.86), and median progression-free survival was 2.7 months in the T-DM1 group and 2.9 months in the taxane group (HR 1.13; *p* = 0.31). T-DM1 had no overall survival or progression-free survival benefits. Furthermore, no clinical or biomarker subgroups, including IHC 3+ subgroups, showed treatment benefits with T-DM1 compared with taxane.

### 2.4. Pertuzumab

Pertuzumab is a humanized anti-HER2 monoclonal antibody that binds to the extracellular domain II (dimerization domain) of HER2, which is different from the trastuzumab binding site [17]. The inhibition of the heterodimerization by pertuzumab leads to the blocking of HER2 activation and HER2-mediated downstream signaling. The CLEOPATRA study showed that pertuzumab significantly improved survival outcomes when added to trastuzumab plus docetaxel in HER2-positive advanced breast cancer [18]. This pertuzumab and trastuzumab-containing chemotherapy is the only regimen recommended for breast cancer by the National Comprehensive Cancer Network guidelines, and is category 1 for the first-line setting at present.

To test whether pertuzumab- and trastuzumab-containing chemotherapy also has survival benefits in HER2-positive advanced gastric cancer, the phase 3 JACOB study was conducted [19]. This was a double-blind, placebo-controlled, randomized trial performed in 30 countries, and 780 patients were randomly assigned to the study treatment, which was the largest study of HER2-targeted therapy for gastric cancer to date. As in the GATSBY study, the “HER2-positive” definition was HER2 IHC 2+ and ISH-positive or IHC 3+, regardless of ISH. Before this study, the phase 2a JOSHUA study was conducted to determine the appropriate dose of pertuzumab for gastric cancer [20]. Patients were randomly assigned (1:1) to groups receiving either pertuzumab (840 mg intravenously) or a placebo every 3 weeks with trastuzumab plus chemotherapy; this was the same regimen and dose as the ToGA study [11]. The primary endpoint was overall survival. At the final analysis, the median overall survival was 17.5 months in the pertuzumab group and 14.2 months in the control group (hazard ratio 0.84; *p* = 0.057). Adding pertuzumab to trastuzumab and chemotherapy numerically improved the overall survival; however, statistical significance was not reached.

## 3. Difficulties in Developing HER2-Targeted Therapy for Gastric Cancer

After the ToGA study, HER2-targeted drugs that are effective for breast cancer successively failed to show survival benefits in gastric cancer. There are clear differences between breast cancer and gastric cancer in HER2-targeted therapy, and hence there are several possible causes of the ineffectiveness of HER2-targeted therapy against gastric cancer.

### 3.1. HER2 Heterogeneity in Gastric Cancer

The most likely reason for HER2 therapy ineffectiveness is the heterogeneity of HER2 overexpression and/or amplification, which many authors highlighted in the discussion sections of their papers [11,16,19]. Generally, one mechanism of resistance to targeted therapy is the heterogeneous expression of the therapeutic target within the tumor [21]. Even in breast cancer, heterogeneous HER2 expression is well known [22] and definitions for HER2 heterogeneity are proposed in the guidelines [23,24]. However, in gastric cancer, the HER2 expression pattern confirmed by IHC is fundamentally heterogeneous and that is why the IHC assessment of HER2 in gastric cancer was changed from breast cancer in the ToGA study and subsequent studies. The results of the quantitative proteomic analysis indicated that the expression levels of the HER2 protein in gastric cancer exhibited significant variation, and in some cases, the levels were undetectable even in tissue samples exhibiting IHC 3+ positivity [25].

HER2 signal dependency may differ between breast cancer and gastric cancer. Breast cancer is classified into six intrinsic molecular subtypes based on gene expression profiles, and the “HER2-enriched” subtype is independent as one of these subtypes [26,27]. Gastric cancer has also been classified into molecular subtypes via comprehensive analysis. Two representative classifications were reported by the Cancer Genome Atlas (TCGA) and the Asian Cancer Research Group (ACRG) [28,29]. TCGA proposed four principal molecular subgroups of gastric cancer: the microsatellite instable (MSI), Epstein–Barr virus (EBV)-associated, chromosomally instable (CIN), and genomically stable groups. Similarly, the ACRG divided gastric cancer into four subtypes: MSI, microsatellite stable (MSS)/*TP53* active, MSS/*TP53* inactive, and MSS/mesenchymal-like. Of note, neither classification independently categorized the “HER2-enriched” subtype. Interestingly, in TCGA classification, HER2 amplification is mainly associated with the CIN subgroup; however, other receptor tyrosine kinase alterations are also included in the same subgroup and partially overlap. Additionally, the EBV subgroup also includes HER2 amplification. These data indicated that in gastric cancer, HER2 alterations are molecularly heterogenous and might not be the independent oncogenic driver; that is, blocking the HER2 pathway might not be enough to kill cancer cells. Thus, HER2-targeted therapy for gastric cancer may require more than just blocking the HER2 pathway.

### 3.2. Definition of HER2-Positive Gastric Cancer

The “HER2-positive” definition is another discussion point. For some other biomarkers for targeted therapies, for instance “EGFR-positive” lung cancer, it is relatively easy to distinguish positive from negative according to whether there is an active *EGFR* gene mutation. Additionally, the effect of EGFR tyrosine kinase inhibitors is clearly different between “EGFR-positive” and “EGFR-negative” lung cancer [30].

However, HER2 is more complicated. HER2 expression has been used as a predictive biomarker of HER2-targeted therapy, but the degree of HER2 expression widely varies from no expression to high expression. In an early clinical trial of trastuzumab against breast cancer, HER2 expression was regarded as positive when more than 25% of tumor cells exhibited characteristic membrane staining for HER2 [31]. In the pivotal phase 2 single-agent and phase 3 combination studies of trastuzumab against breast cancer [8,32], the IHC scoring system was more organized in relation to the HER2 receptor number (no staining (score 0), <20,000 receptors; partial membrane staining with <10% of the cells showing complete membrane staining (score 1+), 100,000 receptors; light to moderate complete membrane staining in >10% of the cells (score 2+), 500,000 receptors; and complete membrane staining in >10% of the cells (score 3+), 2,300,000 receptors) [33]. In these two pivotal studies, HER2 IHC 2+ and 3+ were regarded as “HER2-positive”, but this cutoff was based on biological features, and the results of the studies indicated that patients with a HER2 score of 3+ would gain more benefit from trastuzumab [34]. Therefore, a subsequent study adopted HER2 IHC 3+ as the definition of “HER2-positive” breast cancer [35]. However, these and other data also indicated that trastuzumab had a slight effect against HER2 IHC 2+ breast cancer even though HER2 FISH was negative (HER2:CEP17 ratio < 2.0) [36]. Additionally, recent studies have shown that the intrinsic phenotypes of HER2 IHC 1+/2+ breast cancer overlap among luminal A/B, basal-like, normal-like, and HER2-enriched types [37,38]. The trastuzumab biomarkers of breast cancer would be continuous rather than categorical or binary, and this may be partially independent of genetic phenotypes. Namely, when HER2 expression/amplification is above the artificial cutoff regarding the clinically acceptable benefits of trastuzumab, it is described as “HER2-positive” breast cancer.

From this point of view, the “HER2-positive” definition should vary for each drug, as diverse HER2-targeted therapies necessitate distinct HER2 statuses to attain efficacy. Fortunately, in breast cancer, HER2-targeted therapies have the same “HER2-positive” definition as trastuzumab, but it might be different in gastric cancer. For instance, even though T-DM1 failed to show clinical benefits in ToGA study-based HER2-positive gastric cancer compared with taxane, it seemed that HER2 expression was a predictive biomarker for T-DM1 against gastric cancer [16]. If the cutoff value for HER2 positivity due to HER2 protein levels was set higher, T-DM1 might have demonstrated clinical benefits (however, this is not realistic because of the small number of applicable patients or technical/cost aspects regarding quantifying the amount of protein).

### 3.3. Racial Specificity of Gastric Cancer

Regional differences in gastric cancer are not a major problem but should be noted. Gastric cancer in Asia and the West are biologically different with respect to incidence, etiology, tumor location, and clinicopathological characteristics [39,40]. Asia and the West also have clinical differences with regard to the screening protocols used or treatment modalities, such as surgical approaches or perioperative treatment [41]. Generally, Asian patients tend to have a better prognosis than Western patients [42,43], but it may be a chicken and egg scenario; Asia, especially Japan, tends to use more lines of chemotherapy than the West [44]. These differences complicate the interpretation of the results of global gastric cancer clinical trials.

The AVAGAST study was a global phase 3 study that aimed to evaluate the efficacy of another molecular targeted drug, bevacizumab, against gastric cancer following the ToGA study [45]. Although this study did not show an overall improvement in survival in the whole population, a subgroup analysis indicated that Western patients tended to have a prolonged median overall survival when bevacizumab was added to chemotherapy (11.5 months vs. 6.8 months, hazard ratio 0.63 in Pan America; and 11.1 months vs. 8.6 months, hazard ratio 0.85 in Europe). Conversely, in Asia, the addition of bevacizumab appeared to have no effect on the median overall survival (13.9 months vs. 12.1 months, hazard ratio 0.97). Notably, the numerical median overall survival of Asian patients without bevacizumab was longer than that of Western patients with bevacizumab. The authors pointed out that the difference in the use of the post-progression chemotherapy rate (68% in Asia and 23% other region) was one of the factors that contributed to the different survival outcomes between regions [46]. Even the ToGA study identified similar trends [47]. A subgroup analysis showed that the numerical median overall survival was not different with or without trastuzumab in Japanese patients (15.9 months for trastuzumab plus chemotherapy arm and 17.7 months for chemotherapy arm, hazard ratio 1.00) and that both were longer than the trastuzumab plus chemotherapy arm in the overall population (13·8 months). In the ToGA study, 80.4% in the trastuzumab plus chemotherapy arm and 82% in the chemotherapy arm in Japanese patients received second-line treatment, whereas only 42% in the trastuzumab plus chemotherapy arm and 45% in the chemotherapy arm in the overall population received second-line treatment. Intensive treatment and a long survival in Asia could mitigate the benefits of a new drug in clinical studies, especially in the first-line setting. These trends could also be seen in the latest global phase 3 studies of the PD-1-blocking antibody nivolumab for gastric cancer by comparing Checkmate 649, which was conducted mainly outside of Asia, with the ATTRACTION-4 study, which was conducted in East Asia [48,49,50]. In other words, very powerful HER2-targeted therapy is required to demonstrate a statistically significant overall survival in HER2-positive gastric cancer clinical studies, including in Asia.

In summary, to demonstrate the survival benefits of HER2-targeted therapy for gastric cancer, a drug is required that targets beyond the HER2 pathway-blocking mechanisms and has strong anti-tumor effects, especially for Asian patients, with an appropriate predictive biomarker (“HER2-positive” definition). Recently, a new drug, trastuzumab deruxtecan, has emerged that meets some of these requirements.

## 4. Trastuzumab Deruxtecan, A Novel Anti-HER Agent

### 4.1. Characteristics of Trastuzumab Deruxtecan

Trastuzumab deruxtecan (DS8201) is a novel HER2-targeted ADC. The antibody portion has the same amino acid sequence as trastuzumab, which is similar to T-DM1, but the payload and linker are different to T-DM1. The payload of trastuzumab deruxtecan is a cell membrane-permeable topoisomerase I inhibitor that is 10 times more potent than the active metabolite of the topoisomerase I inhibitor, irinotecan. Additionally, the drug-to-antibody ratio is approximately eight, which is much higher than three for T-DM1 [51,52]. This high cytotoxic payload is conjugated to trastuzumab by a linker that is stable in plasma and selectively cleaved by lysosomal cathepsins that are upregulated in cancer cells [53]. This unique linker and membrane-permeable payload enables trastuzumab deruxtecan to kill neighboring target tumor cells regardless of their HER2 expression [51,54]. This bystander anti-tumor effect is particularly beneficial in tumors with the heterogenous expression of HER2, such as gastric cancer. Of note, T-DM1 has no such a bystander effect [54].

This ADC was initially granted approval by the FDA in 2019 for the treatment of patients afflicted with advanced HER2-positive breast cancer, based on the single-arm phase 2 DESTINY-Breast01 study [55]. The recent phase 3 DESTINY-Breast03 study demonstrated that trastuzumab deruxtecan was superior to T-DM1 for patients with HER2-positive metastatic breast cancer previously treated with trastuzumab and a taxane [56]. The response rate and progression-free survival for the trastuzumab deruxtecan arm in this study implied that single-agent trastuzumab deruxtecan is as potent as pertuzumab- and trastuzumab-containing triplet chemotherapy, the current gold-standard for first-line treatment of advanced HER2-positive breast cancer [18].

Trastuzumab deruxtecan is a promising drug, but there is an adverse event that should be monitored closely: interstitial lung disease. In a pooled analysis of 1150 patients in nine phase 1 and phase 2 clinical studies of trastuzumab deruxtecan monotherapy, drug-related interstitial lung disease occurred in 15.4%, of which, 2.2% of cases were grade 5 [57]. Cox multivariate regression analysis identified seven baseline factors that may contribute to increased drug-related interstitial lung disease: age (<65 years), trastuzumab deruxtecan dose (>6.4 mg/kg), SpO2 (<95%), renal impairment (moderate/severe based on the Cockcroft–Gault formula), presence of lung comorbidities, time since initial diagnosis (>4 years), and enrollment in Japan. Of note, there are scattered data showing that other drug-induced lung damage is also more common in Japan than in other countries [58,59,60].

### 4.2. Trastuzumab Deruxtecan for Gastric Cancer

For advanced gastric cancer, the comparison of this powerful drug with a mechanism of action beyond HER2 inhibition with standard chemotherapies was evaluated in the DESTINY-Gastric01 study [61]. This pivotal phase 2 study was an open label, randomized study in Japan and South Korea that compared trastuzumab deruxtecan with the physician’s choice of chemotherapy in patients with HER2-positive gastric cancer who progressed after two or more previous therapies involving trastuzumab. Before this study, a dose-expansion phase 1 study was conducted, and a suitable dosage of 6.4 mg/kg administered via infusion every three weeks was established for the treatment of gastric cancer, which was higher than that for breast cancer (5.4 mg/kg infusion every 3 weeks) [62]. Patients were randomly assigned (2:1) to groups receiving either trastuzumab deruxtecan or the physician’s choice of chemotherapy (either irinotecan at 150 mg/m^2^ every 2 weeks or paclitaxel at 80 mg/m^2^ on days 1, 8, and 15 every 4 weeks). The primary endpoint was an objective response in an independent central review of patients with high-level HER2-positive (IHC 3+ or IHC 2+ with ISH positivity) disease. Of note, patients with low-level HER2 (IHC 2+ with ISH negativity or IHC 1+) were also included in this study. Overall survival was deemed as a key secondary endpoint, and was evaluated in a hierarchical statistical manner only if the primary endpoint demonstrated significance. A total of 188 patients with high-level HER2-positive disease in the primary cohort underwent randomization. The objective response rate by independent central review (not confirmed) was significantly higher in the trastuzumab deruxtecan group than in the physician’s choice of chemotherapy group (51% vs. 14%, *p* < 0.001). Overall survival was also significantly longer in the trastuzumab deruxtecan group than in the physician’s choice of chemotherapy group (12.5 months vs. 8.4 months; hazard ratio 0.59; *p* = 0.01). Although it was only conducted under the conditions of phase 2 studies in East Asia, trastuzumab deruxtecan was the first HER2-targeted drug to show a survival benefit against gastric cancer since trastuzumab in the ToGA study. Following Japan in 2020, trastuzumab deruxtecan was approved in the United States as a treatment for patients with HER2-positive advanced gastric cancer in 2021 as a result of these efficacy data. Recently, the single-arm phase 2 DESTINY-Gastric02 study demonstrated that trastuzumab deruxtecan was also effective in non-Asian patients in a second-line setting (confirmed objective response rate; 38.0%) [63].

### 4.3. Definition of “HER2 Positivity” in Trastuzumab Deruxtecan

Importantly, this groundbreaking drug could change the definition of “HER2 positivity”. The phase 3 DESTINY-Breast04 study demonstrated that trastuzumab deruxtecan could significantly prolong overall survival compared with the physician’s choice of chemotherapy in patients with HER2 IHC 2+ with ISH-negative or IHC 1+ metastatic breast cancer, which was regarded as HER2-negative by the previous breast cancer definition [64]. As mentioned above, the DESTINY-Gastric01 study also included patients with HER2 IHC 2+ and ISH-negative or IHC 1+ (of note, the IHC scoring system is different to that of breast cancer) gastric cancer. The confirmed objective response rate for the 20 IHC 2+/ISH− patients was 26.3% with a median overall survival of 7.8 months, and the confirmed objective response rate for the 24 IHC 1+ patients was 9.5%, with a median overall survival of 8.5 months [65]. Although it is too early to draw any conclusions because of the small number of patients, these results suggest that trastuzumab deruxtecan has some clinical benefit for “ToGA study-based HER2-negative” gastric cancer.

HER2 mutations may also be a biomarker for trastuzumab deruxtecan. Interestingly, trastuzumab deruxtecan shows clinical benefits in “HER2-mutant” non-small cell lung cancer, regardless of the HER2 expression/amplification status [66,67]. Preclinical data have suggested that active HER2 mutations, regardless of HER2 expression, facilitate its ubiquitination and internalization; these may be important mechanisms underlying endocytosis and the consequent efficacy of trastuzumab deruxtecan [68]. Consistently, preliminary clinical data have suggested that trastuzumab deruxtecan is less effective in patients with HER2-overexpressing non-small cell lung cancer [69,70]. Thus, in non-small cell lung cancer, “HER2 positivity” for trastuzumab deruxtecan currently means “HER2-mutant” rather than “HER2-overexpressing”. With this promising drug, the traditional definition of “HER2 positivity” is being redefined.

### 4.4. Ongoing Clinical Trial of Trastuzumab Deruxtecan for Gastric Cancer

The development of trastuzumab deruxtecan for gastric cancer is more elaborate than the previous HER2-targeted therapies trastuzumab, lapatinib, T-DM1, and pertuzumab. As mentioned previously, an appropriate dose was examined in a phase 1 study, and then the effect was examined in third- or later-line settings in the phase 2 DESTINY-Gastric01 study. Efficacy in the second-line setting will be evaluated in the ongoing phase 3 DESTINY-Gastric04 study (NCT04704934), which compares trastuzumab deruxtecan with paclitaxel plus ramucirumab.

Recently, the initial findings of the first interim analysis of KEYNOTE-811, a phase 3 clinical trial assessing the effectiveness of pembrolizumab or a placebo in combination with trastuzumab and chemotherapy for HER2-positive (IHC 3+ or IHC 2+ with ISH positivity) gastric cancer in the first-line setting, have been reported [71]. Although the primary endpoints of this study are progression-free survival and overall survival, both of which have not been published due to the immaturity of the study, the response rate observed in the study is encouraging, with 74.4% of the patients receiving pembrolizumab demonstrating an objective response compared to 51.9% of those in the placebo group. These results suggest that the use of HER2-targeted therapy in conjunction with immune checkpoint inhibitors could potentially establish a novel standard of care. The phase 1b/2 DESTINY-Gastric03 (NCT04379596) is an ongoing study that aims to evaluate trastuzumab deruxtecan in combination with chemotherapy and the immune checkpoint inhibitors pembrolizumab or durvalumab in first- or second-line settings. If KEYNOTE-811 demonstrates a survival benefit in the pembrolizumab arm and the DESTINY-Gastric03 study shows efficacy and acceptable safety, a clinical trial comparing trastuzumab with trastuzumab deruxtecan alongside the combination of an immune checkpoint inhibitor and chemotherapy will be conducted, the most successful of which may become the gold standard for the future first-line treatment of HER2-positive gastric cancer.

## 5. Novel Anti-HER2 Therapy for Gastric Cancer

Several anti-HER2 drugs are in development, some of which have reached late-stage clinical trials (Table 2). This chapter summarizes the ongoing phase 3 clinical trials of new anti-HER2 therapies.

### 5.1. Zanidatamab and NK026

Zanidatamab (ZW25) is a bispecific monoclonal antibody that is directed against extracellular domain IV, the trastuzumab-binding domain, the dimerization extracellular domain, and the pertuzumab binding domain of HER2. This monoclonal antibody has a mechanism of action beyond HER2 pathway inhibition, including the inhibition of growth factor-independent tumor-cell proliferation and the activation of immune-mediated responses, such as antibody-dependent cellular cytotoxicity, antibody-dependent cellular phagocytosis, and complement dependent cytotoxicity [72]. The promising results of a phase 2 study have been published, showing a confirmed objective response rate of 75% and a median duration of response of 16.4 months in combination with chemotherapy in first-line treatment [73]. The phase 3 HERIZON-GEA-01 study (NCT04276493) is now ongoing [74]. This is a global, randomized, open-label study to compare the efficacy and safety of zanidatamab plus chemotherapy with or without the PD-1-blocking antibody tislelizumab with trastuzumab plus chemotherapy as a first-line treatment for patients with advanced HER2-positive (IHC 3+ or IHC 2+ with ISH positivity) gastric cancer.

KN026 is another bispecific antibody that binds to extracellular domain IV and the dimerization extracellular domain of HER2. The preliminary results of a phase 2 study for second line and later therapy (NCT03925974) showed that the objective response rate was 55.6% and the duration of response was 9.7 months [75]. The ongoing phase 2/3 KN026-CSP-001 study (NCT05427383) will evaluate the safety and efficacy of KN026 in combination with chemotherapy in patients with HER2-positive (IHC 3+ or IHC 2+ with ISH positivity) gastric cancer who have progressed on from or have undergone a trastuzumab-containing regimen in China.

### 5.2. Margetuximab

Margetuximab is an anti-HER2 monoclonal antibody. The binding epitope is the same as trastuzumab, but Fc is engineered to enhance Fc-dependent antitumor activities across all Fc region gamma receptor IIIA (CD16A) genotypes and potentiate innate immunity, adaptive immunity, and the in vitro upregulation of tumor cell PD-L1 expression. This antibody is expected to have a high affinity for immune checkpoint inhibitors. In the phase 1b/2 CP-MGAH22-05 study, the combination of margetuximab and pembrolizumab had a meaningful antitumor effect with an objective response rate of 18% following at least one previous line of therapy with trastuzumab plus chemotherapy, and this antitumor effect was enhanced in HER2 3+ and PD-L1-positive (CPS ≥ 1) tumors with an objective response rate of 44% [76]. The phase 2/3 MAHOGANY study (NCT04499924) is now ongoing to evaluate the effect of margetuximab combination therapy in the first-line setting. This study consists of two cohorts. Cohort A has a single-arm design to evaluate the efficacy of margetuximab in combination with the PD-1-blocking antibody retifanlimab in patients who are both HER2 IHC 3+ and PD-L1+ and are without high microsatellite instability. Cohort B has a randomized, open-label design that will enroll patients who are HER2 IHC 3+ or IHC 2+ and ISH-positive, regardless of PD-L1 status, to evaluate the efficacy of margetuximab plus chemotherapy with retifanlimab or the PD-1 and LAG3 inhibitor tebotelimab [77]. In the interim analysis of cohort A, the objective response rate was 53% and the median duration of response was 10.3 months [78].

### 5.3. Disitamab Vedotin

Disitamab vedotin is an ADC comprising the humanized anti-HER2 antibody hertuzumab conjugated to the microtubule inhibitor monomethyl auristatin E via a cleavable valine–citrulline linker. Similar to trastuzumab deruxtecan, a bystander effect was also observed in this ADC. The single-arm phase 2 RC48-C008 study was conducted in China [79]. Gastric cancer patients with HER2 IHC 2+ or 3+, regardless of ISH status, who were undergoing at least second-line therapy were recruited to this study. More than half of the patients (57.6%) had previously received trastuzumab. Among the 125 eligible patients, the objective response rate was 24.8%, and the progression-free survival and overall survival were 4.1 months and 7.9 months, respectively. The ongoing phase 3 RC48-C007 (NCT04714190) study will further evaluate the efficacy of disitamab vedotin as a second-line or later therapy. This is a multicenter, randomized, open-label study comparing disitamab vedotin with the physician’s choice of chemotherapy, including apatinib, in patients with HER2-overexpressing (IHC3+ or 2+ regardless of ISH status) gastric cancer.

### 5.4. Tucatinib

Tyrosine kinase inhibitors such as lapatinib, afatinib, and poziotinib have not yet been successfully developed for HER2-positive gastric cancer, especially as monotherapy, perhaps because their primary mechanism is to inhibit the HER2 pathway [12,13,80,81,82]. Despite this context, tucatinib, a highly selective small molecule tyrosine kinase inhibitor for HER2, showed promising anti-tumor activity in HER2-positive colorectal cancer in combination with trastuzumab. It is currently being evaluated in the phase 2/3 MOUNTAINEER-02 study (NCT04499924) for HER2-positive gastric cancer in second-line settings after HER2-directed antibody therapy. [83]. The open-label phase 2 study will evaluate the safety of tucatinib in combination with ramucirumab, trastuzumab, and paclitaxel. The phase 3 study is a randomized, double-blind, three-arm study aiming to compare the efficacy of tucatinib and trastuzumab versus a placebo, both in combination with ramucirumab and paclitaxel, and also to evaluate the activity of tucatinib with ramucirumab and paclitaxel [84]. In the phase 3 part, patients who are identified as HER2-positive through blood-based next-generation sequencing are eligible for inclusion, whereas in the phase 2 part, patients who are negative for HER2 via blood-based next-generation sequencing, but positive via a baseline biopsy, are included.

## 6. Summary and Conclusions

The development of HER2-targeted therapies for gastric cancer began well with trastuzumab, with the landmark results of the ToGA trial in 2010. Recently, new types of therapies, including bi-specific antibodies, next-generation tyro-sine kinase inhibitors, CAR-T therapy, and cancer vaccines, are also being developed, thus accelerating the progress of HER2-targeted therapy (Table 2). However, it should be noted that, unlike breast cancer, beneficial treatments targeting HER2 have not appeared easily.

The determination of HER2 positivity is one of the challenges impeding the development of HER2-targeted therapies for gastric cancer. HER2 expression/amplification is not dichotomous but rather exists on a continuous spectrum, necessitating the establishment of a suitable cut-off point. The determination of the valid HER2 status for a particular drug necessitates meticulous consideration, and that status serves as the definition of HER2-positivity for that drug and cancer type. Previous clinical trials for gastric cancer may have disregarded such preliminary exploratory studies.

Targeted therapies against HER2 are being developed for several tumors including non-small cell lung cancer, colorectal cancer, biliary track cancer, and urothelial cancer, taking into account different HER2 statuses [85]. We are also in the process of conducting a clinical trial of trastuzumab deruxtecan for salivary gland cancer with various levels of HER2 expression/amplification (jRCT2011210017). Promising results have already been published for several of these, and thus HER2 will also become a biomarker across various cancer types for targeted therapy [67,86,87,88,89,90]. However, it should be noted that organ specificity and drug specificity should be considered in the development of HER2-targeted therapy. HER2 is a promising therapeutic target for gastric cancer, but effective drugs are still limited. While referring to data from other tumors, it will be necessary to promote developments that are unique to gastric cancer.

## Figures and Tables

**Table 1:** Landmark phase 3 clinical trials of HER2-targeted therapy in gastric cancer.

Clinical Trial	First Reported Year	Drug	HER2 Definition	Phase	Line of Therapy	Intervention (Comparison)	Results
ToGA	2009	Trastuzumab	IHC 3+	P3	First-line	Trastuzumab + chemo	Improvement of median OS
			and/or ISH-positive			(Chemotherapy)	13.8 m vs. 11.1 m, *p* = 0.0046
TyTAN	2013	Lapatinib	ISH-positive	P3	Second-line	Lapatinib + chemo	No difference in median OS
						(Chemotherapy)	11.0 m vs. 8.9 m, *p* = 0.1044
TRIO-013/LOGiC	2013	Lapatinib	IHC 3+	P2/3	First-line	Lapatinib + chemo	No difference in median OS
			and/or ISH-positive			(Chemotherapy)	12.2 m vs. 10.5 m, *p* = 0.91
GATSBY	2016	T-DM1	IHC 3+	P2/3	First-line	T-DM1	No difference in median OS
			or IHC 2+ISH-positive			(Chemotherapy)	7.9 m vs. 8.6 m, *p* = 0.31
JACOB	2017	Pertuzumab	IHC 3+	P3	First-line	Pertuzumab + Trastuzumab + chemo	No difference in median OS
			or IHC 2+ISH-positive			(Trastuzumab + chemo)	17.5 m vs. 14.2 m, *p* = 0.057

**Table 2:** Representative ongoing phase 2 and phase 3 clinical trials of HER2-targeted therapy in gastric cancer.

Types of Treatment	Drug	Clinical Trial	Phase	Line of Therapy	Intervention
Monoclonal antibodies	Margetuximab	NCT04082364	P2/3	First-line	Margetuximab + retifanlimab
		(MAHOGANY)			Margetuximab + retifanlimab + chemo
					Margetuximab + tebotelimab + chemo
					Margetuximab + chemotherapy
					Trastuzumab + chemotherapy
Bispecific antibodies	Zanidatamab	NCT04276493	P3	First-line	Zanidatamab + tislelizumab + chemo
		(HERIZON-GEA-01)			Zanidatamab + chemo
					Trastuzumab + chemo
	KN026	NCT05427383	P2/3	Second-line and beyond	KN026 + Chemo
		(KN026-CSP-001)			Chemo
	Cinrebafusp alfa	NCT05190445	P2	Second-line and beyond	Cinrebafusp alfa + ramucirumab + paclitaxel
					Cinrebafusp alfa + tucatinib
Antibody-drug conjugates	Trastuzumab deruxtecan	NCT04379596	P1b/2	First-line	Trastuzumab deruxtecan + pembrolizumab + chemo
		(DESTINY-Gastric03)			Trastuzumab deruxtecan + chemo
					Trastuzumab deruxtecan + pembrolizumab
					Trastuzumab deruxtecan
					Trastuzumab + chemo
		NCT04704934	P3	Second-line	Trastuzumab deruxtecan
		(DESTINY-Gastric04)			Ramucirumab + paclitaxel
		NCT04989816	P2	Third-line and beyond	Trastuzumab deruxtecan
		(DESTINY-Gastric06)			
	Disitamab vedotin	NCT04714190	P3	Second-line and beyond	Disitamab vedotin +
		(RC48-C007)			chemo
	SBT6050	NCT05091528	P1/2	Second-line and beyond	SBT6050 + trastuzumab + tucatinib + capecitabine
					SBT6050 + trastuzumab + tucatinib
					SBT6050 + trastuzumab deruxtecan
	MRG002	NCT04492488	P1/2	Second-line and beyond	MRG002
Tyrosine kinase inhibitors	Tucatinib	NCT04499924	P2/3	Second-line	Tucatinib + trastuzumab +ramucirumab + paclitaxel
		(MOUNTAINEER-02)			Ramucirumab + paclitaxel
		NCT04430738	P1/2	First-line	Tucatinib + trastuzumab + pembrolizumab + chemo
					Tucatinib + trastuzumab + chemo
	Afatinib	NCT02501603	P2	Second-line	Afatinib + paclitaxel
		NCT01522768	P2	Second-line	Afatinib + paclitaxel
	Pyrotinib	NCT04960943	P2	Second-line and beyond	Pyrotinib + paclitaxel
					Pyrotinib
CAR-T-cell therapy	TAC01-HER2	NCT04727151	P1/2	Third-line and beyond	TAC01-HER2
Vaccine	TAEK-VAC-HerBy	NCT04246671	P1/2	Third-line and beyond	TAEK-VAC-HerBy

## Data Availability

No new data were created or analyzed in this study. Data sharing is not applicable to this article.
